# Lung Transplant for ARDS after COVID-19: Long-Term Outcomes and Considerations about Detrimental Issues

**DOI:** 10.3390/jcm11164754

**Published:** 2022-08-15

**Authors:** Alessandro Palleschi, Stefania Crotti, Anna Mara Scandroglio, Alfredo Lissoni, Evgeny Fominskiy, Lorenzo Rosso, Davide Tosi, Valeria Musso, Francesco Blasi, Andrea Gori, Mario Nosotti

**Affiliations:** 1 Department of Pathophysiology and Transplantation, Università degli Studi di Milano, 20122 Milan, Italy; 2 Thoracic Surgery and Lung Transplantation Unit, Fondazione IRCCS Ca’ Granda–Ospedale Maggiore Policlinico, 20122 Milan, Italy; 3 Anesthesia, Intensive Care and Emergency, Fondazione IRCCS Ca’ Granda–Ospedale Maggiore Policlinico, 20122 Milan, Italy; 4 Department of Anesthesia and Intensive Care, San Raffaele Scientific Institute, Vita-Salute San Raffaele University, 20132 Milan, Italy; 5 Università degli Studi di Milano, 20122 Milan, Italy; 6 Internal Medicine Department, Respiratory Unit and Adult Cystic Fibrosis Center, Fondazione IRCCS Ca’ Granda–Ospedale Maggiore Policlinico, 20122 Milan, Italy; 7 Infectious Disease and Tropical Medicine Unit, Fondazione IRCCS Ca’ Granda–Ospedale Maggiore Policlinico, 20122 Milan, Italy

**Keywords:** COVID-19, lung transplantation, ARDS

## Abstract

During the first outbreak of COVID-19 in Italy, based on the only few cases reported from a Chinese centre at the time, we performed lung transplantation in two patients with irreversible acute respiratory distress syndrome (ARDS) after COVID-19 at our centre. After two years, we report the outcomes of these cases and some considerations. The first patient, an 18-year-old male, is in excellent conditions twenty-four months after surgery. The second patient was a 48-year-old man; his airways were colonized by carbapenemase-producing klebsiella pneumoniae at the time of lung transplantation, and he had previously suffered from delirium and hallucinations in the intensive care unit. His postoperative clinical course was complicated by dysexecutive behaviour and then septic shock; he died 62 days after surgery. The recently reported experience of different transplantation centres has led to the inclusion of irreversible acute respiratory distress syndrome (ARDS) after COVID-19 among the indications for lung transplantation in carefully selected patients. Our results confirm the feasibility and the good long-term outcomes of lung transplantation for COVID-19-associated ARDS. Nonetheless, our experience corroborates the need for careful recipient selection: special attention must be paid to the single-organ dysfunction principle, the evaluation of any neuro-psychiatric disorder, and MDR germs colonization, before listing.

## 1. Introduction

The respiratory system is the preferential target of coronavirus-2 (SARS-CoV-2). The clinical pictures of coronavirus disease 2019 (COVID-19) are extremely diversified, up to the intensive care unit (ICU) admission for acute respiratory distress syndrome (ARDS). The most severely affected patients need extracorporeal respiratory support (ECMO), and some of them are subject to an irreversible evolution of lung disease with massive alveolar damage and pulmonary fibrosis, leading to respiratory failure. A minority of these patients are likely to be considered for lung transplantation. Our centre was among the first to perform lung transplantation for ARDS due to COVID-19 in the Western World in 2020. However, since the beginning of the COVID-19 pandemic, different transplantation centres have reported their early experience with lung transplantation in this setting: the results appeared encouraging, thus leading the transplant community to consider lung transplantation after ARDS due to COVID-19 in carefully selected patients [1,2,3,4]. The exact criteria and contraindications for lung transplantation in these particular circumstances are, however, still under debate, as is the correct timing for transplantation [5], and there are still some concerns with regard to recovery from lung damage and long-term outcomes. Two years after performing lung transplantation in two patients with COVID-19-related ARDS, we report the outcomes and some considerations.

## 2. Presentation of Cases

We retrospectively analysed our first two patients who underwent lung transplantation for irreversible COVID-19-associated ARDS at our centre in Milan, Italy. The study was approved by the ethics committee of Fondazione IRCCS Ca’ Granda Ospedale Maggiore Policlinico Milano.

**Figure 1:**
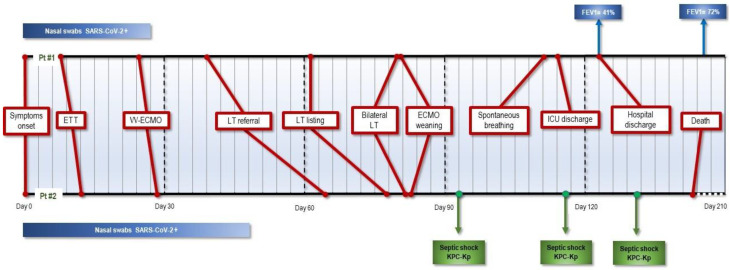
Clinical course timeline. ETT: endotracheal tube; VV-ECMO: veno-venous extracorporeal membrane oxygenation; LT: lung transplantation; ICU: intensive care unit; KPC-Kp: Klebsiella pneumoniae carbapenemase-producing; FEV1: forced expiratory volume in one second.

**Figure 2:**
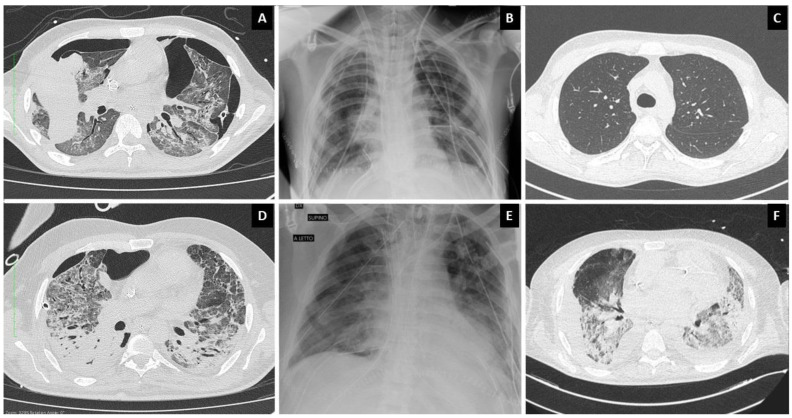
Patients’ imaging. (**A**) Patient-1 preoperative CT; (**B**) patient-1 chest X-ray on post-operative day 1; (**C**) patient-1 CT 4 months after transplantation; (**D**) patient-2 preoperative CT; (**E**) patient-2 chest X-ray on post-operative day 1; (**F**) patient-2 CT before death.

On 18 May 2020, we carried out a bilateral lung transplantation on an 18-year-old male patient; he showed post-operative progressive ample functional recovery [6]. His clinical evolution in the peri-transplant period is summarised in Figure 1. Two years after lung transplantation, the patient is in excellent general condition, with no signs of rejection or anastomotic complications. His FEV1 has reached 77% of the predicted normal value, and at the last six-minute walking test he covered a distance of 512 metres. The second patient was a 48-year-old man who underwent lung transplantation on 8 June 2020. The patient initially seemed to make a good functional recovery, but died 62 days after surgery (Figure 1). Both patients had silent medical histories prior to COVID-19; at listing, they had repeated nasal swabs taken and tested negative for SARS-CoV-2 by molecular and culture investigations. At the time of transplantation, the airways of patient-1 were colonized by Pseudomonas aeruginosa, whereas those of patient-2 were colonized by carbapenemase-producing klebsiella pneumoniae (KPC-Kp); nevertheless, none of the patients showed any sign of active infections. In both cases, we employed our standard protocol for immunosuppression, which consists of prednisone, tacrolimus, and azathioprine, without specific induction therapy. Peri-operative surveillance bronchoscopies were performed at 7 and 15 days, as per our protocol. Once postoperative sedation was reduced, patient-2 showed serious dysexecutive behaviour associated with anxious syndrome that frustrated any rehabilitation effort. During the first days after transplantation, patient 2 had a weak positive SARS-CoV-2 molecular test; the following tests were negative. His postoperative clinical course was complicated by three episodes of septic shock, due to pulmonary and bloodstream KPC-Kp infections, leading to a progressive impairment of lung function. Figure 2 also shows patient-2’s post-operative imaging. After these first two cases, patients were only occasionally referred to our centre and underwent evaluation, but neither were accepted and put on the waiting-list for transplantation.

## 3. Discussion

In February 2020, Italy was hit by the first outbreak of COVID-19, which had a major impact on the Italian healthcare system. An 18-year-old patient with acute respiratory distress syndrome on ECMO was referred to our centre to be evaluated for lung transplantation. Pressed by the young age of patient-1, we took a so-called leap in the dark, because the only lung transplantations for COVID-19-associated ARDS we were aware of at the time were a couple of cases reported by Chen et al. [1]. The first two lung transplantations for ARDS related to COVID-19 in the Occident were performed on the same day at our centre and by the Vienna group [7]. Several arrangements had to be made in our operating theatre in order to keep the staff safe. The number of people in the operating room was reduced to a minimum; they were equipped with full protective clothing, including power air-purifying respirators. The surgery was physically demanding; therefore, the personnel involved changed halfway through transplantation. We showed that transplantations in COVID-19 patients were feasible and that, following appropriate protocols, the procedure could be safe for healthcare staff. The long-term outcomes of our first case two years after surgery also confirmed the validity of lung transplantation for the treatment of COVID-19-related ARDS. Our patients’ early-term outcomes of lung transplantation for respiratory failure after COVID-19 were included in a multicentric case series [2], which was followed by several reports on transplantation after ARDS due to COVID-19. Despite the encouraging results obtained by these groups, some issues, such as criteria for recipient selection, contraindications, and timing, are still to be addressed. Our experience, after two years of follow-up, calls for some considerations. Before putting both patients on the waiting list, thorough evaluations were performed and interdisciplinary discussions were held. The lung damage was considered irreversible in both cases, there were no alternative treatments available, and there were no obvious contraindications to transplantation. The airways of both patients were colonised by bacteria, but there were no signs of active infections. Based on these elements, we decided to include the patients on the waiting list. Our second unsuccessful case, however, brings out possible negative prognostic factors that must be taken into account with particular attention in pre-listing evaluations, together with those warning signs that have recently been summarised [5]. Firstly, we faced a severe dysexecutive behaviour associated with anxious syndrome that led to a total lack of compliance and the inability for patient-2 to fully recover. Psychological distress symptoms are common in ICU patients and are frequently associated with depressive behaviour after discharge. Moreover, neuro-psychiatric disorders have been described in COVID-19 patients [8]. In a series of 58 patients admitted to ICUs for COVID-19-associated ARDS, the prevalence rates of neurological disorders and dysexecutive syndrome were 84% and 33%, respectively [9]. Secondly, a multidrug-resistant (MDR) strain colonized patient-2, causing a life-threatening infection. MDR bacterial infections frequently occur in COVID-19 patients, increasing morbidity and mortality in ICUs. COVID-19 patients in particular display a high rate of MDR superinfection due to many factors, including increased severity of the disease and treatment with immunomodulatory drugs. The implementation of specific protocols for the surveillance and prevention of infectious complications is absolutely necessary [10,11].

After the first two cases, patients who were referred to our centre for evaluation were isolated cases, but none underwent transplantation. In this particular setting, in fact, the optimal timing for referral is essential, and the window of transplant in such cases is very narrow.

## 4. Conclusions

In conclusion, lung transplantation for COVID-19-associated ARDS is possible, and the outcomes of our successful case at the two-year follow-up are excellent. However, especially in the acute setting, special attention must be paid to the “single-organ dysfunction” principle. We advise to carefully evaluate any possible prognostic sign of neuro-psychiatric disorders before listing. Similarly, colonization by MDR germs must be regarded as a possible life-threatening condition.

## Data Availability

The data presented in this study are available on request from the corresponding author. The data are not publicly available due to privacy.
